# FHL2 in arterial medial calcification in chronic kidney disease

**DOI:** 10.1093/ndt/gfae091

**Published:** 2024-04-25

**Authors:** Yuan-Ru Liao, Yu-Cheng Tsai, Tsung-Han Hsieh, Ming-Tsun Tsai, Feng-Yen Lin, Shing-Jong Lin, Chih-Ching Lin, Hou-Yu Chiang, Pao-Hsien Chu, Szu-Yuan Li

**Affiliations:** Division of Nephrology, Department of Internal Medicine, Taipei Veterans General Hospital, Taipei, Taiwan; Department of Medical Research, Taipei Veterans General Hospital, Taipei, Taiwan; Division of Nephrology, Department of Internal Medicine, Taipei Veterans General Hospital, Taipei, Taiwan; Department of Medical Research, Taipei Veterans General Hospital, Taipei, Taiwan; Joint Biobank, Office of Human Research, Taipei Medical University, Taipei, Taiwan; Division of Nephrology, Department of Internal Medicine, Taipei Veterans General Hospital, Taipei, Taiwan; School of Medicine, National Yang Ming Chiao Tung University, Taipei, Taiwan; Division of Cardiology, Department of Internal Medicine, School of Medicine, Taipei Medical University, Taipei, Taiwan; Division of Cardiology, Department of Internal Medicine, School of Medicine, Taipei Medical University, Taipei, Taiwan; Division of Nephrology, Department of Internal Medicine, Taipei Veterans General Hospital, Taipei, Taiwan; School of Medicine, National Yang Ming Chiao Tung University, Taipei, Taiwan; Division of Cardiology, Department of Internal Medicine, Chang Gung Memorial Hospital, Linkou, Taoyuan, Taiwan; College of Medicine, Chang Gung University, Taoyuan, Taiwan; Department of Anatomy, College of Medicine, Chang Gung University, Taoyuan, Taiwan; Graduate Institute of Biomedical Science, College of Medicine, Chang Guang University, Taoyuan, Taiwan; Division of Cardiology, Department of Internal Medicine, Chang Gung Memorial Hospital, Linkou, Taoyuan, Taiwan; College of Medicine, Chang Gung University, Taoyuan, Taiwan; Institute of Stem Cell and Translational Cancer Research, Chang Gung Memorial Hospital, Taiwan; Division of Nephrology, Department of Internal Medicine, Taipei Veterans General Hospital, Taipei, Taiwan; School of Medicine, National Yang Ming Chiao Tung University, Taipei, Taiwan

**Keywords:** *FHL2*, kidney disease, *RUNX2*, vascular calcification, vascular smooth muscle cell

## Abstract

**Background:**

Arterial medial calcification (AMC) is a common complication in individuals with chronic kidney disease (CKD), which can lead to cardiovascular morbidity and mortality. The progression of AMC is controlled by a key transcription factor called runt-related transcription factor 2 (RUNX2), which induces vascular smooth muscle cells (VSMCs) transdifferentiation into an osteogenic phenotype. However, RUNX2 has not been targeted for therapy due to its essential role in bone development. The objective of our study was to discover a RUNX2 coactivator that is highly expressed in arterial VSMCs as a potential therapy for AMC.

**Methods:**

We employed transcriptomic analysis of human data and an animal reporter system to pinpoint four and a half LIM domains 2 (FHL2) as a potential target. Subsequently, we investigated the mRNA and protein expression patterns of FHL2 in the aortas of both human and animal subjects with CKD. To examine the role of FHL2 in the RUNX2 transcription machinery, we conducted coimmunoprecipitation and chromatin immunoprecipitation experiments. Next, we manipulated *FHL2* expression in cultured VSMCs to examine its impact on high phosphate-induced transdifferentiation. Finally, we employed *FHL2*-null mice to confirm the role of FHL2 in the development of AMC *in vivo*.

**Results:**

Among all the potential RUNX2 cofactors, FHL2 displays selective expression within the cardiovascular system. In the context of CKD subjects, FHL2 undergoes upregulation and translocation from the cytosol to the nucleus of arterial VSMCs. Once in the nucleus, FHL2 interacts structurally and functionally with RUNX2, acting as a coactivator of RUNX2. Notably, the inhibition of *FHL2* expression averts transdifferentiation of VSMCs into an osteogenic phenotype and mitigates aortic calcification in uremic animals, without causing any detrimental effects on the skeletal system.

**Conclusion:**

These observations provide evidence that FHL2 is a promising target for treating arterial calcification in patients with CKD.

## Graphical Abstract

**Graphical Abstract:**
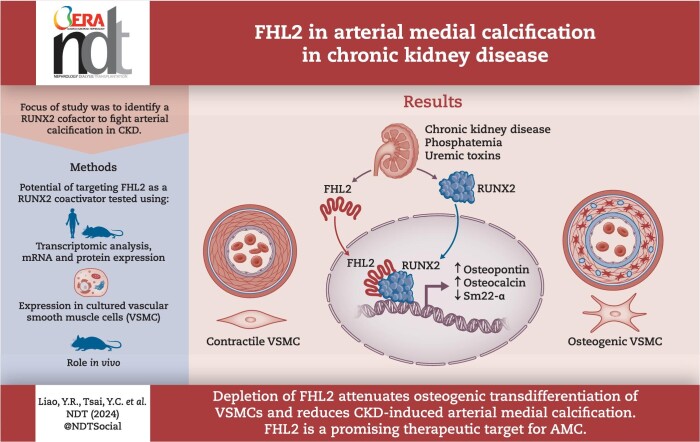

KEY LEARNING POINTS **What was known:**

- Activation of called *runt-related transcription factor 2* (*RUNX2*) in vascular smooth muscle cells (VSMCs) is responsible for their osteogenic transdifferentiation and the resulting arterial calcification in chronic kidney disease (CKD).
- However, targeting *RUNX2* for treatment is unfeasible since it also affects bone health.

**This study adds:**

- This study revealed that *FHL2* is an *RUNX2* cofactor enriched in the cardiovascular system.
- In the context of CKD, it undergos translocation, specifically observed within VSMCs, where it moves from the cytosol to the cell nucleus, functioning as a transcriptional coactivator of *RUNX2*.
- Inhibiting *FHL2* selectively mitigates CKD-induced arterial medial calcification while preserving bone health.

**Potential impact:**

- *FHL2* could be a novel therapeutic target for preventing or mitigating arterial calcification in CKD patients.

## INTRODUCTION

Arterial medial calcification (AMC) is a prevalent pathological feature in patients with chronic kidney disease (CKD), and unfortunately, there are currently no reliable interventions that can effectively prevent or slow the progression of vascular calcification in CKD patients. It is noteworthy that AMC is strongly linked to a significantly elevated mortality rate in this patient population [1, 2]. As such, the development of novel therapeutic strategies aimed at mitigating or reversing AMC in CKD patients is of utmost importance to improve their clinical outcomes and reduce the associated mortality risk [3]. AMC that arises in the context of CKD is an intricate biological process that shares many similarities with bone formation [4]. Specifically, vascular smooth muscle cells (VSMCs) undergo a series of phenotypic changes in response to elevated levels of extracellular phosphate, gradually transforming into cells that resemble osteoblasts and chondroblasts, and actively promoting the mineralization of vascular tissues [4, 5]. As a result, VSMCs lose their contractile phenotype to a more mesenchymal-like character, expressing bone markers Osteocalcin (*OCN*) and Osteopontin (*OPN*) akin to osteoblasts and chondroblasts [6]. During the process of transdifferentiation, VSMCs undergo a shift in gene expression and begin to produce osteogenic transcription factors, including msh homeobox 2 (MSX2), runt-related transcription factor 2 (RUNX2) and SRY-Box 9 (SOX9) [6]. Of these factors, RUNX2 is a pivotal regulator of osteoblast differentiation and exerts a critical influence on the development of AMC. Previous research has demonstrated that reducing the stability of *Runx2* mRNA *in vitro* and enhancing the ubiquitination of RUNX2 protein in VSMCs can mitigate vascular calcification [7–9]. Moreover, VSMC-specific *Runx2* knockout animals are safeguarded against vascular calcification [10, 11], while VSMC-specific *Runx2* overexpression accelerates artery calcification [12]. These findings highlight the important role of *RUNX2* in the development of AMC and provide potential therapeutic targets to fight against vascular calcification. Despite the potential benefits of targeting *RUNX2* to mitigate vascular calcification, its clinical application has been limited by its broad expression in various tissues and involvement in metabolic processes. For instance, studies have demonstrated that mice deficient in functional *Runx2* genes exhibit severe skeletal abnormalities and do not develop normal osteoblasts, highlighting the importance of *RUNX2* in bone development [13]. In humans, loss of *RUNX2* activity is associated with cleidocranial dysplasia and other bone deformities [14, 15]. As such, targeted inhibition of *RUNX2* in VSMCs without affecting other tissues remains a challenge for clinical implication.

Nonetheless, it is possible to modulate the activity of RUNX2 in a tissue-specific manner. The transcriptional activity of RUNX2 is regulated by co-activators, co-repressors, post-translational modification and other transcription factors [16], importantly, cofactors of transcription factors can have cell type-specific functions. The specificity of transcriptional regulation is critical for the proper functioning of different cell types in multicellular organisms. Cofactors can interact with specific transcription factors, leading to the regulation of cell type–specific gene expression programs. These interactions are essential for different cellular processes, including development, differentiation and response to environmental stimuli [17, 18].

The goal of this study was to discover a RUNX2 cofactor that is abundant in VSMCs and examine its potential as a target for therapy. Our findings demonstrate that FHL2 is a co-activator of RUNX2 that is restrictively expressed in the cardiovascular system, and *FHL2* is upregulated in CKD human and animal arteries. We also found that high phosphate induces the translocation of FHL2 from the cytosol into the nucleus, where it binds to RUNX2, enhancing its transcriptional activity and accelerating the transdifferentiation of VSMCs. Finally, we used *Fhl2*-null mice to demonstrate that FHL2 deficiency does not affect normal skeletal development but can protect against AMC in CKD. Our results suggest that *FHL2* may represent a promising therapeutic target for the treatment of vascular calcification in CKD.

## MATERIALS AND METHODS

### Human sample collection and gene expression analysis

As part of our efforts to identify possible cofactors of RUNX2 in vascular smooth muscles, we conducted a search of biological protein–protein interaction databases that include data from both experimentally derived protein–protein interactions and computational predictions. To perform this search, we utilized the highly regarded web data resource known as “STRING” [19] (version 11), which enabled us to survey a wide range of potential RUNX2 cofactors. We excluded any interactions that were only supported by co-occurrence, *in silico* gene fusion or neighborhood expression data. Instead, we focused exclusively on interactions that had been experimentally confirmed. Human aortic tissue samples were obtained from patients with an abdominal aortic aneurysm during the surgery of aortic graft replacement (control group and CKD stage 5 patients group, *n* = 8 each group). This study conformed to the principles outlined in the Declaration of Helsinki and was approved by the institute review board of Chang-Gung Memorial Hospital, Taoyuan, Taiwan [Institutional Review Board (IRB) No. 201701920A3], and all patients provided written informed consent. Aortic valve gene expression profiles were obtained from GEO (http://www.ncbi.nlm.nih.gov/geo) under accession number GSE148219, GSE76712-17 [20, 21]. The gene expression profile among different tissues were obtained from three independent human gene expression datasets [22–24]; these datasets are publicly available resources that provide transcriptomic data for a wide range of human tissues and cell types. The use of publicly available datasets ensures that the results of the study are reproducible and transparent. To analyze the RNA-sequence (RNA-seq) data, we aligned the processed reads to the human reference genome (GRCh37/hg19) using STAR (Version 2.6.1). Transcript quantification from the RNA-seq data was performed using RSEM software (Version 1.3.1).

### Generation of *Fhl2*-null mice

Male mice were used in this study to avoid the potential interference of changing levels of hormones on AMC. To generate *Fhl2*-null mice, C57BL/6 J Fhl2^tm(LacZ-Neo)^, a genomic DNA clone, was isolated from mouse using a 410-bp probe from the 5′ cDNA sequence of *Fhl2*. We then used a cDNA encoding *LacZ* and containing a pGK*Neo* cassette to replace the first exon of the endogenous *Fhl2* gene. This replacement allowed the *LacZ* cDNA to be regulated by the natural *Fhl2* promoter while also disrupting the function of the native *Fhl2* gene [25].

### LacZ staining in *Fhl2*-null mice

To assess β-gal activity via X-gal staining, tissue samples from *Fhl2*-null mice were fixed and stained using established protocols [26, 27]. Staining lasted 6 h at room temperature, and samples were then documented and analyzed with a dissecting microscope.

### Monitoring of aortic calcification in a CKD mouse model

Due to the inherent resistance of mice to develop vascular calcification, traditional CKD models such as 5/6 nephrectomy are not capable of inducing calcification without the addition of supraphysiological levels of vitamin D to their diet [28]. Therefore, we employed the adenine model, which has been demonstrated to elicit gradual onset renal tubular obstruction and typical CKD complications such as renal anemia and arterial calcification [28–30]. Eight-week-old male *Fhl2*-null mice (*Fhl2*–/–, C57BL/6J background) and their wild-type (WT) littermates (*Fhl2*+/+) were randomly allocated to experimental groups consisting of eight animals each, for the purpose of monitoring AMC in a 16-week CKD model. *Fhl2*+/+ and *Fhl2*–/– mice were both divided into two groups. The control cohort received a standard pellet chow regimen, while the CKD cohort received the AIN-93 G diet enriched with 2% phosphorus, alongside magnesium at 0.05%, calcium at 0.51% and vitamin D3 at 1.0 IU/g (TestDiet, USA) plus adenine via oral gavage at a dosage of 50 mg/kg (Sigma, USA), with adenine being dissolved in sterile double-distilled water (ddH_2_O). This dietary and adenine administration protocol was maintained for a duration of 28 days [29, 30]. We assessed serum levels of blood urea nitrogen (BUN), calcium and phosphate by collecting submandibular blood samples at various time points: 0, 4, 8, 12 and 16 weeks for BUN; and 0, 8 and 16 weeks for calcium and inorganic P. The sera were extracted from the supernatant layer after refrigerated centrifugation at 3000 rpm for 10 min. The measurements were conducted using the Fuji Dri-chemi 4000i Chemistry Analyzer. To evaluate the advancement of aortic calcification in mice, micro-computed tomography (micro-CT) scans were conducted at Weeks 0, 8 and 16 following gas anesthesia administration (2%–3% isoflurane). Given the dimensions of the mouse aorta, approximately 1 mm in diameter [31], which is just slightly smaller than the 1.5–2 mm diameter in the distal portion of human coronary arteries [32], we chose to employ General Electric healthcare's software for quantifying calcification levels in the mouse aorta. The Agatston calcium score, which relies on Hounsfield unit density measurements, is a well-established and commonly used method in clinical practice for assessing coronary artery calcification [33]. Following a 16-week period of CKD, the mice were humanly euthanized by subjecting them to a 10-min exposure of CO_2_ while keeping them within their original home cage. The aortas of mice were collected, with some being preserved in formalin and others stored at –80°C for future analysis. All procedures conformed to the guidelines from Directive 2010/63/EU of the European Parliament on the protection of animals used for scientific purposes and the National Institutes of Health Guide for the Care and Use of Laboratory Animals. The institutional animal care and use committee reviewed and approved the animal study (IACUC approval No.: LAC-2020-0378) by the IRB of Chang-Gung Memorial Hospital, Taoyuan, Taiwan.

### Quantitative real time PCR

The RNeasy Mini Kit (Qiagen, USA) was used to extract total RNA from aortic tissue and cultured cells. cDNA was synthesized using the cDNA Archival Kit (Life Technologies, USA) with 1 μg RNA. Quantitative real time PCR (qRT-PCR) was conducted on the ViiA 7 System (Life Technologies, USA) using gene-specific primers and SYBR Green Master Mix. The ΔΔCt method was used to normalize the data, with 18S rRNA serving as the housekeeping gene. The primer sequences used in this study are listed in Supplementary data, Table S1.

### Immunohistochemistry and immunofluorescence staining

For immunostaining, the tissue sections were first deparaffinized in xylene and then gradually rehydrated using a series of alcohol concentrations until they reached distilled water. To retrieve the antigen, microwave irradiation in 1 mM ethylenediaminetetraacetic acid (EDTA) was applied. And for cellular fixation and permeabilization, the cultured cells samples were treated with 4% paraformaldehyde and Triton X-100, respectively. Tissue sections or cells samples were then incubated with 10% bovine albumin for 1 h to reduce nonspecific background staining. Sections or cells were then incubated overnight at 4°C with primary antibodies and the secondary antibodies for 1 h at room temperature. The vendors and catalog numbers of antibodies used in this study are listed in Supplementary data, Table S2. We employed immunofluorescence staining to assess alterations in FHL2 protein expression within the aortas of mice with CKD. A group of 12 WT mice was administered an adenine along with AIN-93G diet and then sacrificed 28 days later. Another 12 mice received standard chow served as a control group. Autofluorescence was diminished by TrueVIEW® Autofluorescence Quenching Kit (SP-8500-15, Vector Laboratories, USA). The images were collected with a Zeiss LSM880 confocal microscope. The Von Kossa stain is employed to identify abnormal calcium deposits using a standard protocol. This staining method relies on the conversion of calcium salts to silver salts, visually manifested as black entities.

### Cell culture and *in vitro* model of vascular calcification

We used a mouse aortic smooth muscle cell (MOVAS) cell line (ATCC #CRL-2797) for our experiment. This decision was made due to the significant variability in gene expression characteristics that can be observed in primary cells sourced from different mice or regions of the same aorta. Additionally, as the induction of calcification required a 14-day high phosphate treatment that necessitated splitting the cells several times, we could not use primary culture cells since they have a limited lifespan and can only be passaged a few times. MOVAS cells, incubated at 37°C with 5% CO_2_, were maintained in the standard growth medium consisting of high-glucose Dulbecco's Modified Eagle Medium (Gibco, USA) supplemented with 10% fetal bovine serum (Gibco, USA), 500 μg/mL G418 (Gibco, USA) and 1% penicillin–streptomycin (P/S) (Gibco, USA), and the standard growth medium was replenished every 2–3 days as previously described [34]. To generate stable MOVAS cell line with *Fhl2* knockdown, we employed a lentivirus system using short hairpin RNA (shRNA) to knockdown *Fhl2* in MOVAS cells and obtained sh*Fhl2*-221 and sh*Fhl2*-354 from the Taiwan Academia Sinica core facility (TASCF). To produce the lentivirus, we mixed knockdown plasmids with two packaging plasmids, pCMV-dR8.91 and pMD2.G, in a ratio of 1:0.9:0.1. The total amount of plasmid was 2 µg. We were co-transfected the total plasmids into HEK293T cells in 2 mL complete medium using TurboFect transfection reagent (Thermo Scientific, USA). The ratio of DNA/Turbofect was 1:3. We collected the virus suspension by filtering the culture media through Nalgene 0.22-μm filters at 48 h post-transfection. We added the virus suspension (200 μL) to MOVAS cells to transduce the lentivirus. Transduced cells were selected using puromycin (2 μg/mL, Gibco, USA) for a week. Synbio-Tech (synbio-tech.com) generated the lentivector pCDH-CMV-MCS-EF1α-Puro carrying *Fhl2* cDNA. *Fhl2* overexpression in MOVAS cells was achieved through lentivirus transduction, followed by puromycin selection at 2 µg/mL for a week. Control cells were infected with an empty vector. Additionally, we employed shRNA to knock down *Runx2*, with shRunx2 obtained from TASCF. To induce calcification, MOVAS cells were cultured in the standard growth medium plus with 3.0 mM sodium dihydrogen phosphate (NaH_2_PO_4_) (Sigma, USA) and maintained at 37°C in a 5% CO_2_ incubator for 14 days.

### Cytosolic and nuclear protein extraction

Cells were scraped by iced phosphate-buffered saline. After cells were centrifuged at 1000*g* for 5 min, the first supernatant was removed, and then added cytosol extraction buffer [10 mM Hepes pH 8.0, 1 mM MgCl2, 1 mM EDTA pH 8.0, 10 mM KCl, 0.5 mM dithiothreitol (DTT), 4 μg/mL Leupeptin, 20 μg/mL Aprotinin, 0.2 mM phenylmethylsulfonyl fluoride (PMSF) and 0.5% Igepal CA-630] and mixed by vortex for 15 s, then centrifuged at 3000*g* for 15 min at 4°C before collecting supernatant for cytosolic protein. The pellets were the added to nuclear extraction buffer (10 mM Hepes pH8.0, 1 mM MgCl_2_, 0.25 mM EDTA pH 8.0, 400 mM KCl, 0.5 mM DTT, 4 μg/mL Leupeptin, 20 μg/mL Aprotinin, 0.2 mM PMSF and 10% glycerol) and vortexed for 15 min, then centrifuged at 15 000*g* for 30 min at 4°C, and the supernatants were collected and stored at –80°C for further experiment.

### Coimmunoprecipitation

FHL2 and Runx2 were coimmunoprecipitated by a commercial kit (26 149, Pierce™ Co-Immunoprecipitation Kit, Thermo Scientific, USA) according to the manufacturer's protocol. Anti-FHL2 antibody (MBL #K0055-3, Japan) and anti-Runx2 antibody (MBL #D130-3, Japan) used for coimmunoprecipitation (Co-IP) were both 7.5 μL/mg protein.

### Western blot

Cells and aorta samples were homogenized in RIPA buffer supplemented with protease and phosphatase inhibitors (ApexBio, USA). A 20-µg protein sample was denatured and then subjected to SDS-PAGE, followed by immunoblotting with antibodies against the target genes. Blots were visualized using an ECL Kit (Visual Protein, Taiwan) and the BioSpectrum 600 Imaging System (UVP, USA). Western blot experiments were independently replicated at least three times, and densitometry results from western blotting were quantified with ImageJ software. For information regarding the antibodies used in western blot, please refer to Supplementary data, Table S2.

### Chromatin immunoprecipitation to validate protein–DNA interactions

We employed the Magna chromatin immunoprecipitation (ChIP) A/G kit (Millipore #17-10 085, USA) for conducting ChIP assay. To initiate chromatin crosslinking, 1 × 10^7^ MOVAS cells were incubated with 1% formaldehyde in the standard growth medium for 10 min. The cross-linked DNA was then fragmented to 200–1000 base pairs in length using sonication, and 1 × 10^6^ cell equivalent of lysate was utilized for each IP reaction. The IP reaction was carried out overnight at 4°C using anti-Fhl2 (MBL #K0055-3, Japan) antibody, anti-Runx2 antibody (MBL #D130-3, Japan) or mouse immunoglobulin G. Subsequently, the DNA was purified and analyzed using PCR to target RUNX2 binding site on osteocalcin (*Ocn*) and osteopontin (*Opn*). The assay was performed using the following primer sequences: *Ocn* promoter: 5′-AGTCTCCTATTGTGGCCTCTCG-3′ and 5′-CCTCCAGCGTCCAGTAGCATT-3′; *Opn* promoter: 5′-AACCACAAAACCAGAGGAGGAAG-3′ and 5′-GAATGCCTGCCGCAGAAGACTGC-3′; and *U6* (as negative control for IP): 5′-CACAGACTTGTGGGAGAAGC-3′ and 5′-GGGTGAGTTTCCTTTTGTGC-3′.

### Statistical analysis

All values are expressed as mean ± standard deviation. Statistical analysis of the data was performed using GraphPad Prism 6 software. The two-tailed Student's *t*-test was used to compare the means between two groups, whereas two-way analysis of variance (ANOVA) and Bonferroni *post hoc* test were used to compare the means among multiple groups. A level of *P* < .05 is considered statistically significant.

## RESULTS

### FHL2 is a key candidate for regulating RUNX2 activity in vascular smooth muscles

**Figure 1:**
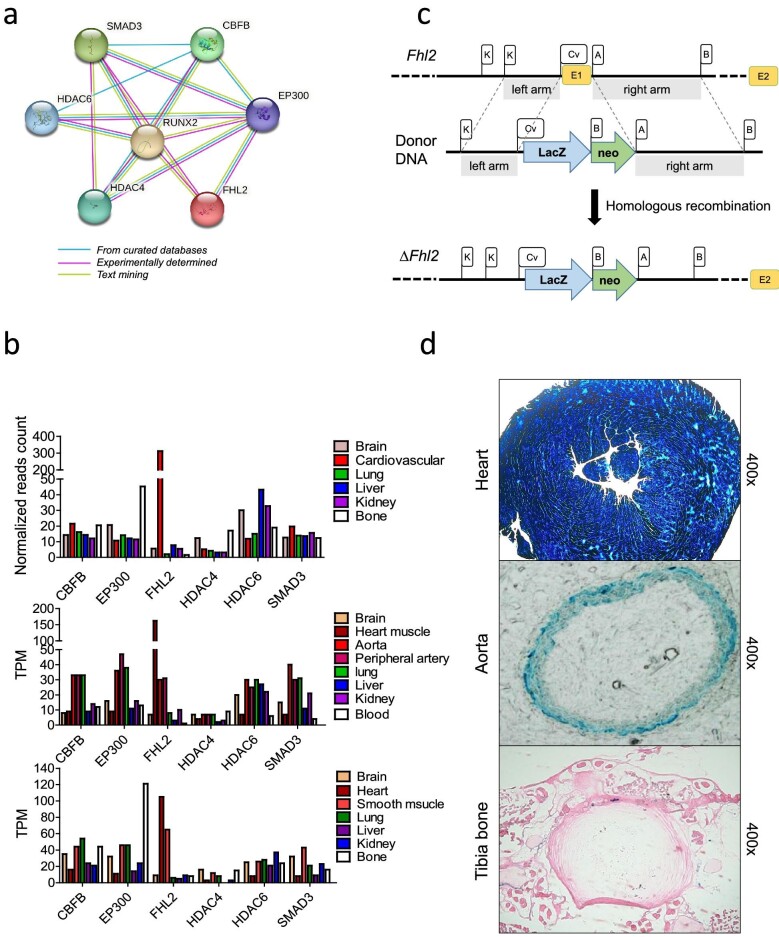
FHL2 is a potential vascular smooth cell enriched RUNX2 cofactor. (**a**) STRING analysis identifies six potential interacting proteins of RUNX2. (**b**) Tissue expression pattern of the six RUNX2-interacting proteins in human transcriptome databases. (**c**) The schematic diagram depicts the process used to generate a *Fhl2*-null *LacZ* reporter mouse model. (**d**) Analysis of *LacZ* staining in the heart, aorta and bone of *Fhl2*-null mice (*n* = 2). The positive staining results from the integration of the *Escherichia coli* gene *LacZ* into the *Fhl2* locus within the genome of reporter mice, indicating a robust activation of *Fhl2* transcription in both cardiomyocytes and arterial smooth muscle cells.

Figure 1a illustrates the six potential RUNX2-interacting proteins in STRING protein–protein interaction database, which are CBFB, EP300, FHL2, HDAC4, HDAC6 and SMAD3. To further explore the significance of these proteins in the context of our research, we evaluated their tissue distribution throughout the human body. This was accomplished by analyzing three independent RNA-seq datasets [22–24], which offer tissue-specific gene expression patterns obtained from clinical human samples. Figure 1b demonstrates that among the six RUNX2 cofactors, only FHL2 displays high expression selectively in the cardiovascular system, unlike the other five proteins which lack this selective expression pattern. It is noteworthy that FHL2 exhibits very low expression in bone tissue, suggesting an improbable impact on osteoblast RUNX2 activity and bone development.

To experimentally confirm *FHL2* expression *in vivo*, we used the *LacZ* reporter system to generate a *Fhl2*-null reporter mouse, in which exon 1 of the *Fhl2* gene was replaced by the *LacZ* reporter gene (Fig. 1c). This mouse strain transgenically expresses the *LacZ* reporter gene, which encodes β-galactosidase (β-gal), under FHL2 transcriptional control. Because the enzymatic activity of the β-gal reporter can be visualized at cellular resolution using X-gal, it is a powerful tool to monitor *Fhl2* expression *in vivo*. Our findings indicate high expression of Fhl2 in cardiomyocytes and arterial smooth muscle cells, while its expression is low in the tibial bone of adult animals (Fig. 1d). In summary, the protein–protein interaction data and our *in vivo* reporter system has led us to identify FHL2 as a promising candidate for regulating RUNX2 activity in vascular smooth muscles and potential therapeutic target for cardiovascular diseases.

### Increased *FHL2* expression in CKD mouse model and human aorta samples

**Figure 2:**
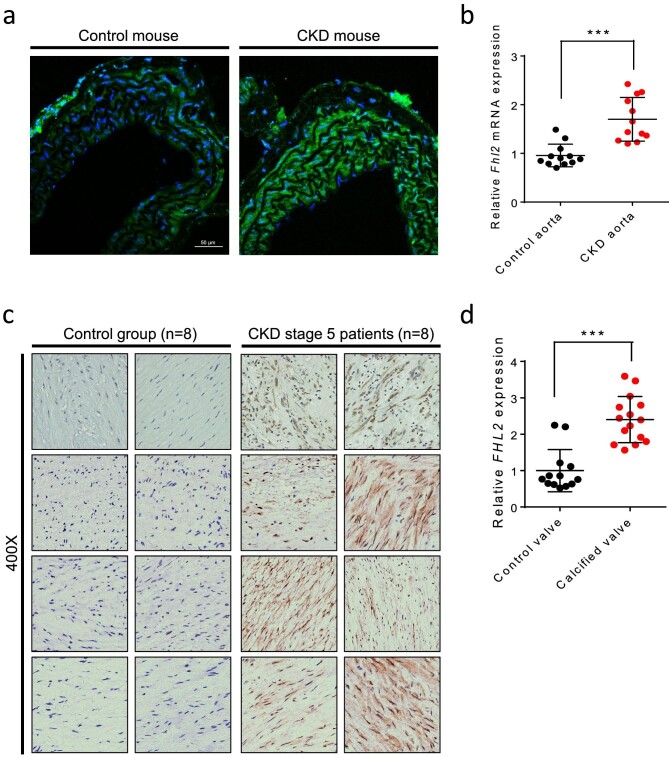
FHL2 is upregulated in arterial vascular smooth muscle in CKD. (**a**) A representative immunofluorescent staining of FHL2 was performed on both control (*Fhl2*+/+) and CKD mouse (*Fhl2*+/+ CKD, 28 days of adenine) aorta using identical laser power and gain settings on a confocal microscope (*n* = 12 each group). FHL2: green color; DAPI: blue color. (**b**) *Fhl2* mRNA level in control (*Fhl2*+/+) and CKD (*Fhl2*+/+ CKD, 28 days of adenine) aorta as measured by qRT-PCR (*n* = 12 each group). (**c**) Representative figures of FHL2 immunohistochemistry staining in control and CKD human aortas (*n* = 8 each group). FHL2: brown color; hematoxylin (nuclear): blue color. (**d**) *FHL2* gene expression in control and CKD human aortic valves. ***P < .001; two-tailed Student's *t*-test.

We next investigated the expression level of *FHL2* in the aortas of CKD patients and mice. We induced CKD in mice by adenine gavage, which causes kidney tubular obstruction and insidious onset of kidney failure. We then analyzed *FHL2* expression by immunofluorescent staining and qRT-PCR. Consistent with our *LacZ in vivo* reporter assay, we observed elevated expression of FHL2 in the muscle layer of the mouse aorta. When applying identical laser power and gain settings, mice with adenine diet-induced CKD for 4 weeks exhibited a more pronounced Fhl2 signal compared with control animals (Fig. 2a). qRT-PCR analysis confirmed that *Fhl2* expression was 1.7-fold higher (*P* < .001) in CKD animals (Fig. 2b). We also utilized immunohistochemistry to analyze FHL2 expression in 16 human aorta samples. Consistent with our animal experiment results, immunohistochemical staining revealed a significant upregulation of FHL2 in aortic VSMCs of CKD patients (Fig. 2c). Additionally, RNA sequence analysis of human aortic valves showed that calcified valves had 2.2 times higher *FHL2* expression (*P* < .001) (Fig. 2d). Taken together, these findings suggest that FHL2 is upregulated in the cardiovascular system in CKD.

### FHL2 subcellular localization is regulated by phosphate and linked to RUNX2 signaling in VSMCs

**Figure 3:**
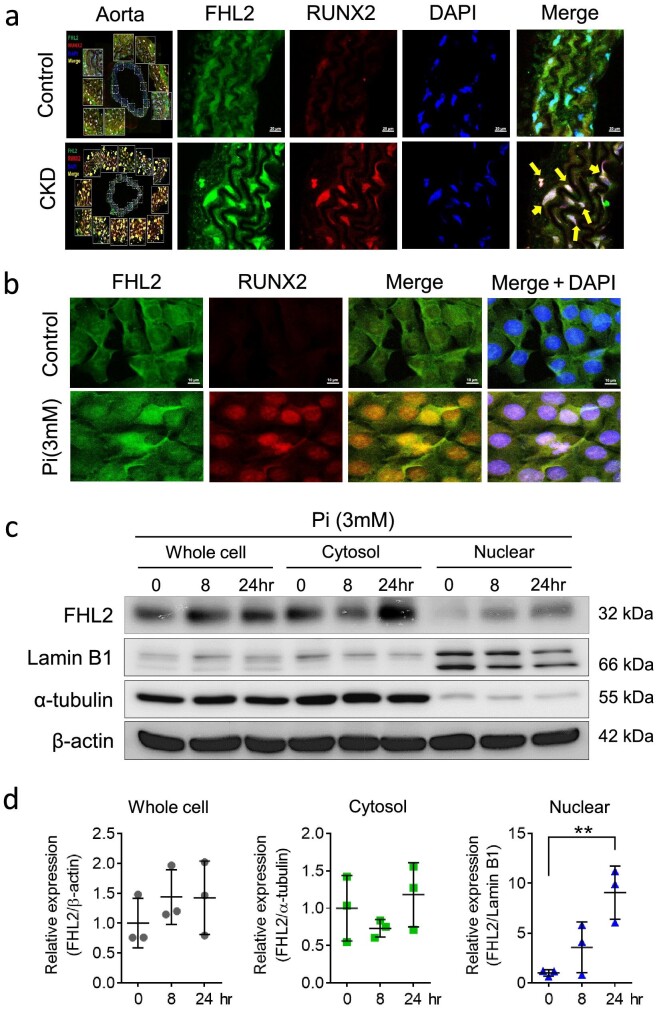
In CKD, FHL2 undergoes translocation to the nucleus and exhibits colocalization with RUNX2 in VSMCs. (**a**) Immunofluorescence staining was performed to examine the distribution of *Fhl2* and *Runx2* in mouse aortas. In control mice, *Fhl2* was found predominantly in the cytosol of arterial VSMCs, while *Runx2* staining showed a negative result. However, in CKD-affected aortas, VSMCs cells exhibited transdifferentiation, displaying strong nuclear staining for *Runx2*, indicating an activated RUNX2 pathway. Additionally, *Fhl2* underwent translocation from the cytosol to the nucleus and co-localized with *Runx2* in the CKD aorta (*n* = 3 for each group). (**b**) Immunofluorescence staining of cultured VSMCs reveals the activation of *Runx2* and nuclear translocation of *Fhl2* upon exposure to phosphate (the experiment was conducted in triplicate). (**c**) Subcellular protein extraction followed by western blot analysis of phosphate-induced *Fhl2* translocation from the cytosol to the nucleus. (**d**) Quantifying changes in *Fhl2* expression at the subcellular level in response to phosphate stimulation. **P < .001; two-tailed Student's *t*-test.

FHL2 is expressed in a cell and tissue-specific manner, and its biological function varies significantly across different cell types and even subcellular locations [35]. Several studies have shown that stimuli can modulate the subcellular distribution of FHL2 [27, 36, 37]. We observed a distinct difference in FHL2 subcellular distribution between control and CKD aorta of mice. Control mice had cytosolic FHL2 expression in a sheet-like pattern in the medial layer of the aorta with no RUNX2 signaling, whereas in CKD animals, FHL2 staining was predominantly nuclear, with strong RUNX2 present in the nucleus (Fig. 3a). As high serum phosphate is the key trigger for RUNX2 activation and VSMCs osteogenic transdifferentiation [38], we examined the effect of phosphate on FHL2 subcellular localization. To induce osteogenic transdifferentiation, cultured MOVAS cells were exposed to sodium dihydrogen phosphate (3 mM) for 14 days [39]. As previously reported, phosphate activated RUNX2 signaling, as evidenced by strong immunofluorescence RUNX2 staining in the cell nucleus. Notably, phosphate also caused FHL2 to translocate from the cell cytosol to the nucleus (Fig. 3b). To further confirm FHL2 subcellular distribution, we performed nuclear/cytoplasmic protein extraction. Our findings revealed that under basal conditions, FHL2 expression predominantly localized within the cytosolic compartment, whereas elevated phosphate levels were found to trigger its migration from the cytosol to the nucleus (Fig. 3c and d). These findings indicate that phosphate stimuli play a regulatory role in FHL2 subcellular localization and that this localization may closely linked to the RUNX2 signaling pathway in VSMCs.

### FHL2 and RUNX2 form a protein–protein interaction and cooperatively regulate osteogenic gene expression in VSMCs

**Figure 4:**
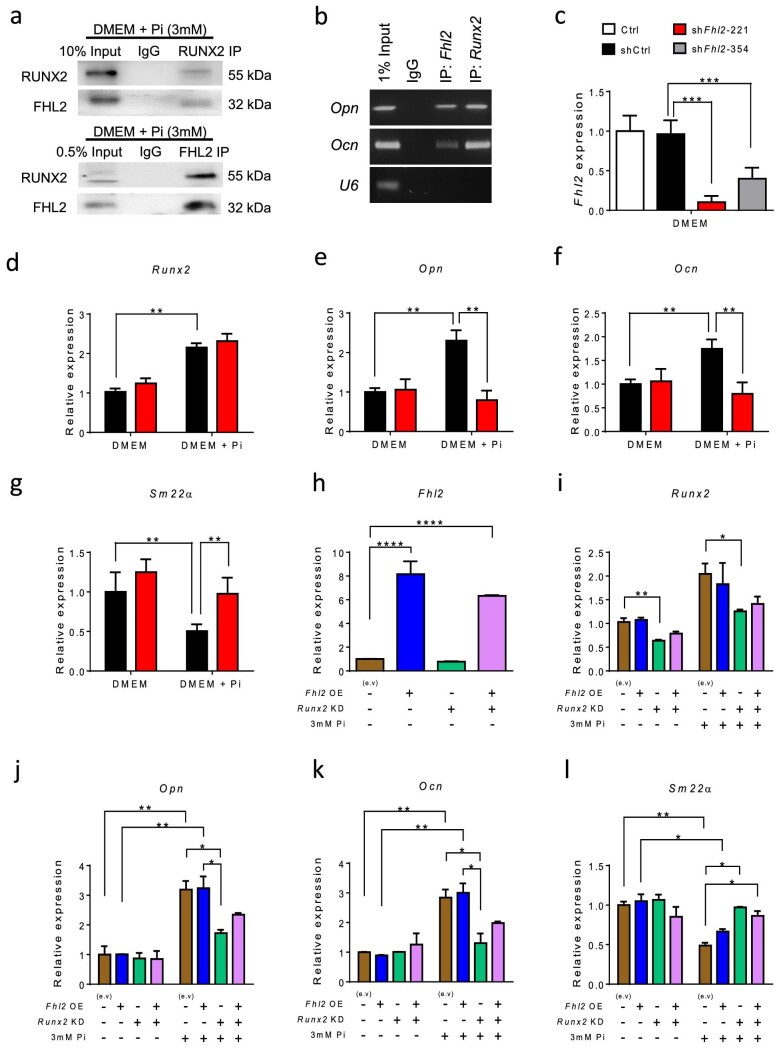
FHL2 is a transcriptional co-activator of RUNX2. (**a**) Co-IP experiments yielded conclusive evidence indicating the existence of a robust structural protein–protein interaction between *Fhl2* and *Runx2* in cultured MOVAS cells. (**b**) Chromatin Immunoprecipitation reveals *Fhl2* and *Runx2* binding at genomic regions of interest, *Fhl2* specifically binds to the *Runx2* binding sites located at the *Ocn* and *Opn* genes. (**c**) Knockdown efficiency of *Fhl2* shRNAs in MOVAS cell line. (**d**–**g**) The influence of *Fhl2* knockdown in *Runx2, Opn, Ocn* and the smooth muscle marker *Sm22α* in phosphate stimulated MOVAS cells. (**h**) The efficiency of *Fhl2* overexpression in MOVAS cells before and after phosphate stimulation. (**i**–**l**) The effects of *Fhl2* overexpression and *Runx2* knockdown on markers of osteocyte and VSMC before and after phosphate stimulation (*n* = 3 each group). *P < .05; **P < .01; ***P < .001; two-way ANOVA followed by Bonferroni post *hoc* test.

After observing the translocation of FHL2 into the nucleus of VSMCs and its co-localization with RUNX2 in CKD, we aimed to investigate whether FHL2 interacts with RUNX2, considering that proteins typically function as part of a dynamic network rather than in isolation. Specifically, we sought to explore the possibility of a physiological protein–protein interaction between FHL2 and RUNX2 within the nucleus of VSMCs. To confirm the structural interaction between RUNX2 and FHL2 proteins, we conducted a Co-IP experiment with the MOVAS cells treated with 3 mmol/L sodium dihydrogen phosphate. Our findings revealed that RUNX2-specific antibodies could indirectly capture FHL2 protein, and conversely, FHL2 antibodies were able to capture RUNX2 protein. These results provided confirmation of the interaction between the two proteins (Fig. 4a). To investigate the regulatory role of FHL2 and RUNX2 and to verify the binding of this protein complex to specific DNA target sequences of RUNX2, we performed ChIP assays targeting specific genomic regions. Our findings revealed that FHL2 specifically binds to the RUNX2 binding site within the *Ocn* and *Opn* genes. This indicates that FHL2 functions as a cofactor within the FHL2 transcriptional complex, suggesting its involvement in the regulation of gene expression (Fig. 4b). We next investigated the possibility that FHL2 influences the RUNX2 pathway signaling by using short hairpin RNA to knockdown *FHL2* in cultured cells. Among the two shRNAs (sh*Fhl2*-221 and sh*Fhl2*-354) tested, sh*Fhl2*-221 was chosen for the following experiments because of its superior silencing effect (Fig. 4c). We then compared phosphate-induced VSMC transdifferentiation in control and *Fhl2* knockdown cells. Consistent with previous findings, high phosphate upregulates the expression of *Runx2* and its downstream osteogenic genes, including *Ocn* and *Opn*, while suppressing the mature smooth muscle marker, *Sm22α*. Under normal culture conditions, knockdown of *Fhl2* did not affect *Runx2, Ocn, Opn* or *Sm22α* expression. In high phosphate culture medium, knockdown of *Fhl2* did not affect *Runx2* expression, but significantly attenuated the upregulation of osteoblast genes *Opn* and *Ocn*, while conserving *Sm22α* expression (Fig. 4d–g). We also used sh*Fhl2*-354 for the same experiments and obtained comparable results (Supplementary data, Fig. S1). We then proceeded to overexpress *Fhl2* in MOVAS cells. Surprisingly, this did not lead to osteo-transdifferentiation. In contrast, the knockdown of *Runx2* markedly reduced phosphate-induced osteo-transdifferentiation (Fig. 4h–l). These results indicate that FHL2 primarily acts as a transcriptional cofactor for RUNX2 in MOVAS cells. Therefore, an increase in FHL2 expression alone may not enhance the signaling of RUNX2 unless RUNX2 is already activated and located in the cell nucleus. In summary, our findings provide evidence that FHL2 plays a role in the RUNX2 transcription complex within VSMCs, acting as a coactivator for RUNX2. Additionally, our findings indicate that the process of VSMC-osteoblast transdifferentiation induced by phosphate could be attenuated by inhibiting *Fhl2*.

### FHL2 deletion attenuates aortic calcification in CKD mice

**Figure 5:**
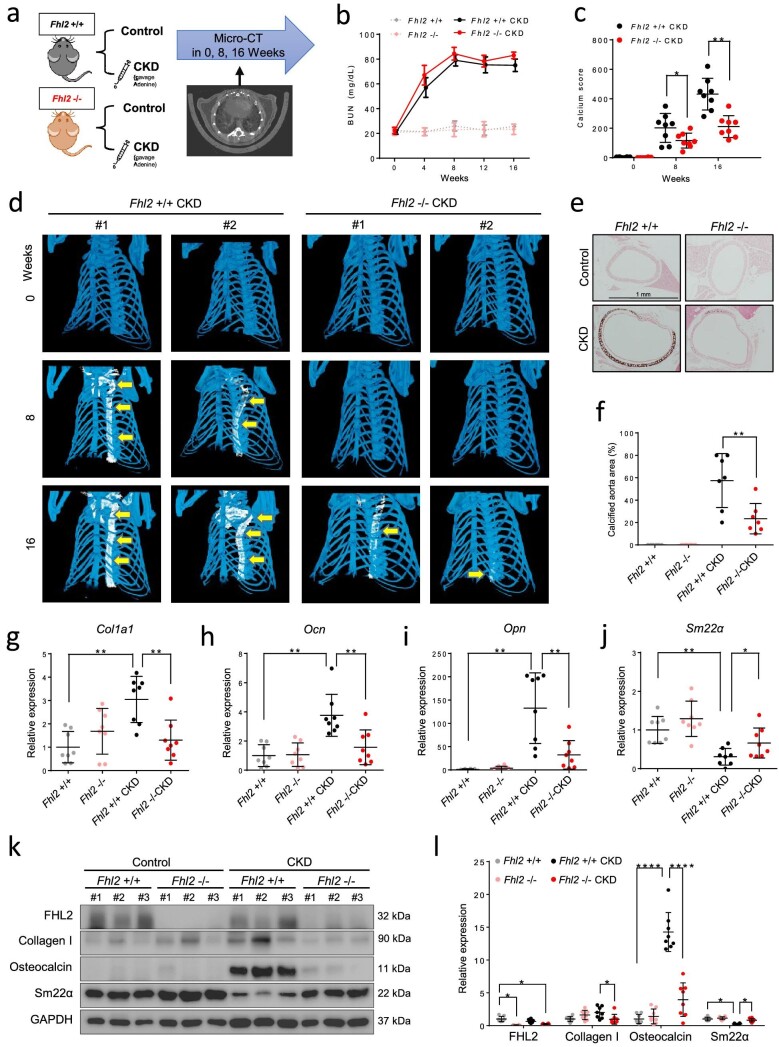
*Fhl2*-null mice are protected from CKD induced arterial calcification. (**a**) The schematic diagram of CKD model and micro-CT scan in *Fhl2*+/+ and *Fhl2*–/– mice. (**b**) Serial changes of serum BUN level in the four groups. (**c**) Quantification of micro-CT scan analysis in aorta calcification. (**d**) Representative figures of aorta calcification at different time points. (**e**) A representative figure of Von Kossa staining in mouse aorta in the four groups. (**f**) Quantification of aorta calcification area. (**g**–**j**) mRNA expression of *Col1a1, Ocn, Opn* and *Sm22α* in mouse aorta. (**k**) Representative western blots of protein expression in mouse aortas. (**l**) Quantification of western blots (*n* = 8 each group). *P < .05; **P < .01; ***P < .001; ****P < .0001. Two-way ANOVA followed by Bonferroni post *hoc* test.

Subsequently, an animal model was employed to investigate the role of *Fhl2* in AMC *in vivo*. We induced CKD in 8-week-old *Fhl2*-null mice (*Fhl2*–/–) and their littermate wild type controls (*Fhl2*+/+) by administering adenine and a high phosphate diet for a period of 4 months. Throughout the study, all mice underwent non-contrast medium-enhanced micro-CT scans at Weeks 0, 8 and 16 to assess the extent of aorta calcification. The progressive elevation of serum BUN levels in the two adenine groups confirmed the development of CKD (Fig. 5a and b). Both CKD groups exhibited comparable levels of serum calcium and hyperphosphate levels (Supplementary data, Table S3). Micro-CT scan enables clear differentiation of calcified vessels (white) and skeleton (blue). At the beginning of the study, both groups showed no aorta calcification and were identical in terms of body size and bone density. In the 8 weeks, aorta calcification was detected in *Fhl2*+/+ CKD group (yellow arrows). By 16 weeks, most *Fhl2*+/+ CKD mice had developed severe, pipe-like aortic calcification with a high calcium score, whereas *Fhl2*–/– CKD mice had significantly less aorta calcification (Fig. 5c and d). At the end of 16 weeks, the mice were euthanized, and aorta tissue was carefully removed for calcium staining and gene expression analysis. Consistent with micro-CT scan results, the aorta from the *Fhl2*+/+ CKD group exhibited diffused medial layer calcification, as evidenced by Von Kossa stain. In contrast, the *Fhl2*–/– CKD mice showed significantly less calcification area (Fig. 5e and f). qRT-PCR analysis revealed that in the *Fhl2*+/+ CKD group, the expression of osteogenic genes, including *Col1a1, Ocn* and *Opn*, were significantly upregulated, whereas the expression of the vascular smooth muscle cell marker gene *Sm22α* was downregulated (Fig. 5g–j). Interestingly, *Fhl2*–/– mice exhibited a similar baseline expression of these genes compared with their *Fhl2*+/+ littermate controls. However, the gene expression pattern was significantly less altered in *Fhl2*–/– CKD compared with *Fhl2*+/+ CKD animals, indicating that FHL2 plays a critical role in CKD-induced VSMC transdifferentiation (Fig. 5g–j). The western blot analysis of the mouse aortas confirmed the same protein expression changes (Fig. 5k and l). To summarize, these findings underscore the vital involvement of FHL2 in *in vivo* arterial medial calcification, and its absence mitigates arterial medial calcification in CKD.

## DISCUSSION

Identifying the crucial intracellular pathways governing phosphate-induced vascular calcification can serve as a basis for devising successful therapeutic strategies to impede the advancement of vascular calcification in CKD [6, 7]. These approaches have promising potential to alleviate the substantial morbidity and mortality linked with AMC in CKD patients. In this study, a new strategy of targeting the RUNX2 transcriptional machinery in CKD-induced vascular calcification is introduced. Our data show that FHL2 plays a crucial role as a cofactor of RUNX2 in VSMCs and is upregulated in CKD human and mouse aortas. Upon entering the cell nucleus, FHL2 works together with RUNX2 to activate gene expression, leading to VSMC transdifferentiation. Since FHL2 exhibits high expression levels in the cardiovascular system but low in bones, its deficiency does not have an impact on bone development. However, it does lead to a significant reduction in CKD-induced AMC. These results suggest that targeting FHL2 could be an effective strategy to combat CKD-induced vascular calcification.

In contrast to the elevated FHL2 levels observed in the aorta of 4-week CKD animals and the aorta of CKD patients, our cultured MOVAS cells did not show a significant increase in Fhl2 expression when exposed to phosphate. We hypothesize that factors beyond phosphate, such as CKD-related cytokines, angiotensin II or transforming growth factor-beta (TGF-β), may be responsible for stimulating *FHL2* in VSMCs *in vivo* [27]. To our surprise, in the *Fhl2*+/+ CKD group, the FHL2 protein level did not significantly increase. This lack of increase is likely attributed to the severe aortic calcification induced by 16 weeks of CKD in the study animals. This condition led to pronounced VSMC-osteogenic transdifferentiation, resembling osteoblasts, ultimately resulting in reduced FHL2 expression. These results suggest that while the upregulation of FHL2 may initiate VSMC transdifferentiation in early CKD, its role diminishes in advanced vascular calcification.

While transcription factors bind specific DNA sequences to provide specificity for gene regulation, they typically lack enzymatic activities to modify chromatin structure and regulate mRNA production, which is usually accomplished by specific proteins known as coactivators [40]. Coactivators are proteins that do not bind DNA but are required for transcriptional activation [41]. In osteoblasts and chondrocytes, RUNX2 alone is not capable of driving bone development. It depends on coactivators EP300 and CBFB [42] to activate downstream gene expressions for normal osteoblast maturation [42, 43]. Apart from its role as a master regulator of osteoblast differentiation, RUNX2 has also been implicated in various other cellular processes, including cell cycle regulation [44], tumorigenesis [45] and arteriosclerotic disease [46]. However, the cofactors of RUNX2 that function beyond osteoblast differentiation remain poorly understood. In this study, we have for the first time demonstrated that FHL2 acts as a coactivator of RUNX2 in VSMCs and promotes arterial calcification in the context of CKD.

The Four-and-a-half LIM (FHL) proteins are a family of proteins that are characterized by four complete LIM domains, which are protein-interaction motifs involved in linking proteins with both the actin cytoskeleton and transcriptional machinery. The LIM domains are preceded by an N-terminal half LIM domain. There are four members of the FHL family: FHL1, FHL2, FHL3 and FHL5. These proteins share 44%–59% sequence conservation with each other but have different tissue expression patterns [47]. FHL proteins typically exist in multi-protein complexes and can either promote or prevent interactions between other proteins. The FHL proteins have been implicated in a wide range of cellular processes, including development, differentiation and disease [48–50]. FHL2 functions as an early marker of cardiogenic cells and serves as a cardiac vascular-specific LIM protein in adults. While FHL2 is not essential for normal cardiac development, it does alter the hypertrophic response to beta-adrenergic stimulation [51], establishing its protective role in hypertrophic settings [52]. FHL2 has been reported to act as a corepressor of hypoxia-inducible factor 1 (HIF-1) [53, 54] and vascular endothelial growth factor [55] in endothelial cells, thereby suppressing neo-angiogenesis. Additionally, FHL2 has been found to interact with bone morphogenetic protein receptor type 2 (BMPRII) to modulate VSMC proliferation [56], indicating that FHL2 has diverse roles in vascular biology. In the current study, we discovered that FHL2 functions as a cofactor of RUNX2 in VSMCs, and the absence of FHL2 attenuates CKD-induced AMC. Importantly, FHL2 is not essential for heart development and function, as evidenced by similar lifespans of Fhl2-null mice and WT animals [25], suggesting that FHL2 inhibition is a safe strategy for preventing vascular calcification in CKD. Of note, all LIM domain–only proteins, including FHL2, lack a DNA binding domain, functioning solely as transcriptional cofactors [57]. Consequently, increased FHL2 expression could not proportionally enhance RUNX2 signaling if RUNX2 is not already activated and expressed in the VSMCs nucleus. Our *in vitro* experiments show that as a co-factor, FHL2 alone cannot upregulate Runx2 function. Therefore, FHL2 up-regulation may have no consequence, and a meaningful impact would only result from its inhibition.

The process of osteogenic transdifferentiation in VSMCs is a phenomenon that promotes arterial calcification and appears to commence prior to the deposition of minerals. Our results demonstrate that FHL2 translocates to the nucleus and co-activates RUNX2 activity during this critical process. Interestingly, our findings are consistent with previous studies suggesting that matrix mechanics and the RHO signaling pathway may regulate FHL2 nuclear translocation [37, 58, 59]. Notably, Gunther *et al*. employed pulldown assays with bacterially expressed mutant proteins to elucidate the interaction between FHL2’s C-terminal LIM domains 3 and 4, and RUNX2’s RUNT, AD3 and RD domains [60]. These results, in conjunction with our findings, provide further insight into the molecular mechanisms underlying the regulation of osteogenic transdifferentiation in VSMCs. The transdifferentiation of VSMCs in AMC is a complex and multifactorial pathological mechanism. In addition to RUNX2 signaling, WNT/β-catenin [61, 62], SIRT-1 [63], nuclear factor-κB [64, 65] and TGF-β [66] all have their roles in osteo-inductive signaling within VSMCs. Interestingly, previous studies have shown that FHL2 interacts with these transcription factors in different cell types [27, 53, 67–69]. Further investigations are warranted to elucidate whether FHL2 interacts with these transcription factors in the nucleus of VSMCs and its involvement in these pathways in AMC.

CKD patients have an increased cardiovascular disease risk because of uremic toxin accumulation, accelerated arterial calcification and dysregulated lipid metabolism. Given the pleiotropic nature of FHL2 and its involvement in numerous cellular pathways, the effect of FHL2 depletion on cardiovascular diseases must be assessed through *in vivo* studies. Notably, FHL2 depletion has shown beneficial effects in mitigating high fat diet–induced atherosclerosis [70, 71] and arterial stenosis [72]. However, it is worth noting that FHL2 depletion has also been associated with increased inflammation in an animal model of carotid ligation [73]. These contradictory outcomes may stem from the divergent pathophysiological contexts of acute inflammation induced by carotid ligation versus the chronic vascular diseases such as atherosclerosis and arterial calcification. The findings from our study suggest that FHL2 inhibition could potentially be considered for preventing vascular calcification in patients with CKD; however, further studies are needed to validate its effects on vascular biology and overall health.

In conclusion, our investigation reveals that FHL2 is expressed exclusively in the cardiovascular system and is upregulated in arterial VSMCs in the context of CKD. Our data demonstrate that FHL2 serves as a transcriptional coactivator of RUNX2, with evidence of their physical interaction confirmed via Co-IP and ChIP experiments. Importantly, FHL2 depletion specifically attenuates CKD-induced AMC without affecting bone development and maturation. These findings suggest that FHL2 may represent a novel therapeutic target for AMC.

## Supplementary Material

gfae091_Supplemental_File

## Data Availability

All data generated or analyzed during this study are included in this published article and its supplementary information files.
