# Supplementary material for: FHL2 in arterial medial calcification in chronic kidney disease
Source: Nephrol Dial Transplant. 2024 Apr 25;39(12):2025–39. doi: 10.1093/ndt/gfae091 (PMC11596093; doi:10.1093/ndt/gfae091)
Supplement: gfae091_Supplemental_File [file gfae091_supplemental_file.pdf]

FHL2 as a Key Regulator of RUNX2-Mediated Arterial Medial Calcification in Chronic Kidney Disease

Yuan-Ru Liao[1,2†], Yu-Cheng Tsai[1,2†], Tsung-Han Hsieh[3], Ming-Tsun Tsai[1,4], Feng-Yen Lin[5], Shing-Jong Lin[5], Chih-Ching Lin[1,4], Hou-Yu Chiang[6,7,8], Pao-Hsien Chu[6]*, Szu-Yuan Li[1,4]*

Supplementary data

Figure1. The influence of *Fhl2 (shFhl2*-354*)* knockdown in *Runx2*, *Opn*, *Ocn,* and the smooth muscle marker *Sm22α* in phosphate-stimulated MOVAS cells.

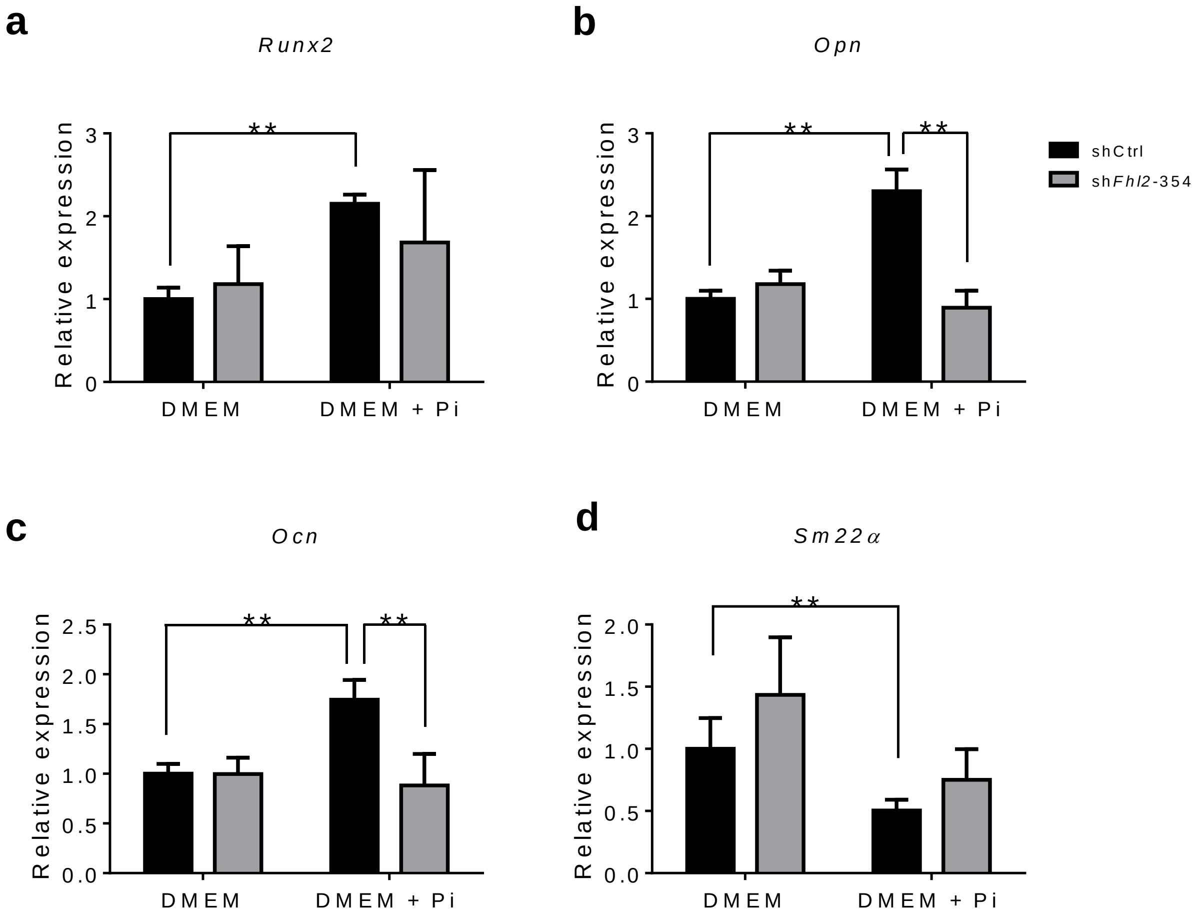

Table 1. qRT-PCR Primers sequences list:

Gene Name	Forward (5'->3')	Reverse (5'->3')
m-*18S rRNA*	GCA ATT ATT CCC CAT GAA CG	GGC CTC ACT AAA CCA TCC AA
m-*Fhl2*	GCC CTG CTA TGA GAA GCA GT	CTG TGC ACA CAA AGC ATT CC
m-*Runx2*	CCC TA ACT CTG CAC CAA GT	TGG CTC AGA TAG GAG GGG TA
m-*Ocn*	GCG CTC TGT CTC TCT GAC CT	GCC GGA GTC TGT TCA CTA CC
m-*Sm22α*	GAT ATG GCA GCA GTG CAG AG	TCT TAT GCT CCT GGG CTT TC
m-*Opn*	CTC CAA TCG TCC CTA CAG TCG	GGC ATC AGG ATA CTG TTC ATC AGA
m-*Col1a1*	CTG GCG GTT CAG GTC CAA T	TTC CAG GCA ATC CAC GAG C

Table 2. Antibodies list:

Name	Producer/Catalog No.	Titer
anti-FHL2	MBL/K0055-3	WB: 1:1000; IF: 1:100; IHC: 1:200
		IP: 7.5μl/mg protein
anti-RUNX2	MBL/D130-3	WB: 1:1000; IF: 1:100;
		IP: 7.5μl/mg protein
anti-Collagen I	Santa Cruz/sc-293182	WB: 1:200
anti-Osteocalcin	Proteintech/23418-1-AP	WB: 1:1000
anti-SM22α	Proteintech/10493-1-AP	WB: 1:1000
anti-β actin	GeneTex/GTX109639	WB: 1:10000
anti-GAPDH	GeneTex/GTX100118	WB: 1:10000
anti-α Tubulin	GeneTex/GTX112141	WB: 1:10000
anti-Lamin B1	GeneTex/GTX103292	WB: 1:1000
Anti-Mouse	Jackson Immuno/115-035-003	WB: 1:10000
Anti-Rabbit	Jackson Immuno/111-035-003	WB: 1:10000
IgG	Cell signaling/2729	IP: 2μl/mg protein

Table 3. Serum calcium (Ca) and phosphate (Pi) levels in *Fhl2*+/+ and *Fhl2*-/- Control and CKD mice

Group	mg/dL	0 week	8 week	16 week
Fhl2+/+ Control	Ca	8.5 ± 0.4	8.9 ± 0.7	8.9 ± 0.8
	Pi	9.7 ± 1.4	8.7 ± 2.1	8.5 ± 0.8
Fhl2-/- Control	Ca	9.2 ± 1.0	9.1 ± 0.6	9.3 ± 0.6
	Pi	10.5 ± 1.3	8.7 ± 1.7	9.2 ± 1.0
Fhl2+/+ CKD	Ca	9.5 ± 1.0	8.3 ± 0.8	10.2 ± 1.2
	Pi	10.2 ± 1.0	13.3 ± 2.8	14.7 ± 0.5
Fhl2-/- CKD	Ca	9.2 ± 1.0	8.7 ± 0.4	10.7 ± 2.2
	Pi	10.3 ± 0.9	11.5 ± 2.7	15.0 ± 0.2

n=8 each group
